# State-Level Medicaid Expenditures Attributable to Smoking

**Published:** 2009-06-15

**Authors:** Brian S. Armour, Eric A. Finkelstein, Ian C. Fiebelkorn

**Affiliations:** Centers for Disease Control and Prevention; RTI International, Research Triangle Park, North Carolina; RTI International, Research Triangle Park, North Carolina

## Abstract

**Introduction:**

Medicaid recipients are disproportionately affected by tobacco-related disease because their smoking prevalence is approximately 53% greater than that of the overall US adult population. This study estimates state-level smoking-attributable Medicaid expenditures.

**Methods:**

We used state-level and national data and a 4-part econometric model to estimate the fraction of each state's Medicaid expenditures attributable to smoking. These fractions were multiplied by state-level Medicaid expenditure estimates obtained from the Centers for Medicare and Medicaid Services to estimate smoking-attributable expenditures.

**Results:**

The smoking-attributable fraction for all states was 11.0% (95% confidence interval, 0.4%-17.0%). Medicaid smoking-attributable expenditures ranged from $40 million (Wyoming) to $3.3 billion (New York) in 2004 and totaled $22 billion nationwide.

**Conclusion:**

Cigarette smoking accounts for a sizeable share of annual state Medicaid expenditures. To reduce smoking prevalence among recipients and the growth rate in smoking-attributable Medicaid expenditures, state health departments and state health plans such as Medicaid are encouraged to provide free or low-cost access to smoking cessation counseling and medication.

## Introduction

Medicaid is a means-tested entitlement program that provides health care coverage to approximately 58 million low-income Americans, many of whom would otherwise be uninsured (1,2). The Medicaid program is jointly financed by the federal and state governments. In 2005, depending on a state's average personal income level, the federal Medicaid matching rate ranged from 50% to 83% (1). The Congressional Budget Office estimates that federal Medicaid expenditures were $191 billion in 2007 (3). Assuming an average Medicaid matching rate of 57%, program expenditures for all 50 states and the District of Columbia are projected to have exceeded $144 billion in 2007 (4,5). By 2018, total federal Medicaid spending is projected to be $445 billion, and assuming a 57% matching rate, total state Medicaid spending is projected to exceed $335 billion (3).

As a percentage of state budgets, Medicaid expenditures increased from 8% in 1985 to 21.5% in 2006, surpassing elementary and secondary education as the largest single budget item (2,5). Medicaid expenditures are expected to consume an ever-increasing share of state budgets, and many states will have difficulty meeting their Medicaid commitments without cutting other state-funded programs (1,5,6). In response to growing concern among state governments, the chairman and vice-chairman of the National Governors Association, in testimony before the US Senate Finance Committee, recommended placing a greater emphasis on disease prevention as a means to contain rising Medicaid costs (6).

Tobacco-cessation programs are effective in lowering the prevalence of cigarette smoking and its consequent serious and costly medical conditions, including pregnancy-related complications, heart disease, respiratory illness, and several types of cancer (7-9). Tobacco-cessation programs should target Medicaid recipients because smoking prevalence in the adult Medicaid population is approximately 53% greater than that of the overall US adult population (34.5% vs 22.6% in 2006) (10).

We used data from the Medical Expenditure Panel Survey (MEPS) and the Behavioral Risk Factor Surveillance System (BRFSS) to update previous estimates of Medicaid smoking-attributable medical expenditures at the state level (11). These estimates might assist state health departments and Medicaid in formulating effective smoking-cessation polices to help reduce the high prevalence of cigarette use among their recipients.

## Methods

### Data

We used the 2001 and 2002 MEPS to develop a model that predicts smoking-attributable medical expenditures for the Medicaid population. MEPS is a nationally representative survey of the civilian, noninstitutionalized population that quantifies each participant's total annual medical spending, including expenditures from public- and private-sector health insurers and out-of-pocket payments. The data also include information about each participant's source of health insurance (eg, any evidence of Medicaid coverage during the year) and sociodemographic characteristics (such as race/ethnicity, sex, and education). Information about MEPS is available at www.meps.ahrq.gov/mepsweb/.

The MEPS sampling frame is drawn from participants in the National Health Interview Survey (NHIS). NHIS is a nationally representative survey that collects data on selected health topics. Although MEPS does not capture information on smoking, self-reported smoking variables are available for a subset of adult NHIS participants (the Adult Sample File) and can be merged with MEPS data. We used responses to the question "Have you smoked at least 100 cigarettes in your entire life?" to differentiate between ever smokers and nonsmokers. We excluded from the analysis sample respondents with missing data on smoking variables (≈1% of respondents aged ≥18 years and all respondents aged <18 at the time of the NHIS interview) and those who did not receive Medicaid coverage. Our final MEPS-NHIS population included 1,588 adults with weighting variables that allowed us to generate nationally representative estimates of the adult, civilian, noninstitutionalized Medicaid population (Table 1).

Before constructing our national model, we used the Medical Care component of the Consumer Price Index to inflate all MEPS annual medical spending data to 2004 dollars.

### State-level representative data

The BRFSS is a state-based telephone survey of the adult (aged ≥18), noninstitutionalized population that tracks health risks in the United States. The most recent BRFSS surveys do not allow for stratifying participants by type of health insurance. This information was, however, available before 2001. Therefore, we used 1998-2000 BRFSS data to predict state-level medical expenditures for the Medicaid population. Information about BRFSS is available at www.cdc.gov/BRFSS/. Although BRFSS does not collect medical expenditure data, it includes information about each participant's smoking status, insurance status (before 2001), and sociodemographic characteristics (such as race/ethnicity, sex, and education). Because these variables match those from MEPS-NHIS, we were able to construct an expenditure prediction model with MEPS-NHIS data and use the results to generate expenditure estimates for smokers and nonsmokers on the basis of state-representative population characteristics of BRFSS participants.

As we did with our MEPS-NHIS restrictions, we excluded those with missing smoking data (≈1%) and those who did not receive Medicaid coverage. Our final BRFSS population included 16,201 adults with weighting variables that allowed us to generate state-representative estimates of the adult, noninstitutionalized Medicaid population (Table 1).

Estimating state-specific smoking-attributable medical expenditures for the Medicaid population involved 3 steps. First, we used MEPS-NHIS data to create a model that predicts annual medical expenditures for Medicaid recipients as a function of smoking status, body weight, and sociodemographic characteristics. Second, we used state-representative BRFSS data and results from our MEPS-NHIS national model to estimate the fraction of medical expenditures for Medicaid recipients that was attributable to smoking for each state. Third, we multiplied these fractions by previously published estimates of state-specific Medicaid expenditures to compute smoking-attributable Medicaid expenditures for each state. These steps are described in detail below.

### MEPS-NHIS national model

We used a 4-part regression model to predict annual medical expenditures for each MEPS-NHIS Medicaid recipient. The 4-part regression approach was pioneered by authors of the RAND Health Insurance Experiment to control for several unique characteristics of the medical expenditures distribution and is now commonly applied to medical expenditures data (12,13). The model estimates predicted expenditures by using the following functional form: *EXP* = *Pr*(*C* × *EXP_IP_* + [1 − *C*]*EXP_NIP_*), where *EXP* represents predicted annual expenditures; *Pr* represents the predicted probability of positive medical expenditures during the year and is estimated with a logistic regression model; *C* represents the conditional probability of positive inpatient expenditures, given positive expenditures, and is estimated with a logistic regression model; *EXP_IP_* represents ordinary least squares (OLS)-predicted medical expenditures, given positive inpatient expenditures during the year; and *EXP_NIP_* represents OLS-predicted medical expenditures, given positive expenditures but no inpatient expenditures.

All OLS regression models are estimated on the logged expenditure variable to adjust for the skewness in annual expenditures (mean annual expenditures are significantly greater than the median). Logged expenditures are converted back to expenditures by using the homoscedastic smearing factor (14).

Including dummy variables that indicate smoking status (ever smoked set equal to 1 and the referent group, never smoked, set equal to 0) in each regression model allowed us to quantify the effect of smoking on annual medical expenditures. In addition to smoking status, all regressions controlled for other variables assumed to influence annual medical expenditures, including self-reported body weight, sex, race/ethnicity, age, region of residence, education, and marital status. Regression models were estimated by using SUDAAN version 8 (RTI International, Research Triangle Park, North Carolina) to control for the complex survey design used in MEPS-NHIS. Table 2 presents results from the 4-part regression model.

### BRFSS state-level estimates

We used the coefficient estimates from the MEPS-NHIS models to predict annual medical expenditures for each BRFSS Medicaid recipient. To do this, we multiplied each person's characteristics (the independent variables) by his respective coefficients generated from the 4 MEPS-NHIS regression models and combined the results with the equation above. Using the BRFSS weighting variables and each person's predicted medical expenditures, we computed total predicted medical expenditures for each state's Medicaid population.

We estimated smoking-attributable medical expenditures as the difference between predicted expenditures for ever smokers and predicted expenditures for nonsmokers, leaving all other variables unchanged. This method allowed us to isolate the effect of smoking while maintaining any other population characteristics that may contribute to higher annual medical expenditures among smokers.

For the Medicaid population in each state, the percentage of aggregate medical expenditures attributable to smoking was calculated by dividing aggregate predicted expenditures attributable to smoking by total predicted expenditures for adult Medicaid recipients in each state. Because BRFSS is limited to adults, our results should be interpreted as the fraction of adult medical expenditures that are attributable to smoking among adults in each state.

### Estimating total and public-sector expenditures

For a variety of reasons, including the lack of data on institutionalized populations, MEPS national spending estimates (and state-level spending estimates based on MEPS) underestimate actual US health care spending (15). Therefore, to quantify annual adult smoking-attributable medical expenditures for each state, we multiplied our state-by-state smoking-attributable fractions by published estimates of 2001 state-specific Medicaid expenditures, available from the Centers for Medicare and Medicaid Services (16). We used 2001 because it is the most recent year that annual, state-specific Medicaid expenditure estimates are available. To match our regression population, we limited Medicaid expenditures to those accrued by adult recipients (≥18 years). We then inflated medical expenditure estimates to 2004 by using a national adjustment factor (1.31). This adjustment factor, calculated as the ratio of 2004 projected expenditures (actual expenditures not yet available) to 2001 actual expenditures, was based on data from Centers for Medicare and Medicaid Services National Health Expenditure Accounts, generally considered the standard for measuring annual health care spending (17).

## Results

State-specific estimates of smoking prevalence among Medicaid recipients vary considerably across states and range from 35% (Mississippi) to 80% (New Hampshire) (Table 3). Nationally, approximately 11% (95% confidence interval, 0.4%-17.0%) of adult Medicaid expenditures are attributable to smoking. At the state level, smoking-attributable fractions range from 6% (New Jersey) to 18% (Arizona and Washington).

Smoking-attributable medical expenditures in the adult Medicaid population total $22 billion. State-level smoking-attributable medical expenditures among adult Medicaid recipients range from $40 million (Wyoming) to $3.3 billion (New York) (Figure).

**Figure 1:**
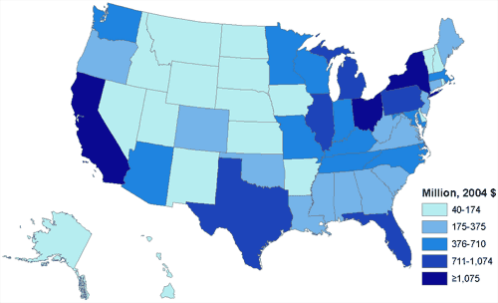
State-by-state distribution of Medicaid smoking-attributable medical expenditures.

## Discussion

The 2000 Public Health Service (PHS) clinical practice guideline for treating tobacco dependence recommends individual, group, and telephone counseling, as well as 5 medications (18). Treating tobacco dependence is more cost-effective than commonly covered preventive services such as mammography or treatment of mild to moderate hypertension (19). In 2002, however, only 10 states reported using the 2000 PHS guideline to design treatment benefits and programs for Medicaid recipients or to train Medicaid health care providers. Moreover, only 5 states required providers or health plans to document tobacco use in patients' medical charts, and only 2 states offered all counseling and pharmacotherapy treatments recommended by the guideline to their Medicaid recipients (20).

The growth rate in Medicaid expenditures has led the National Governors Association to propose a bipartisan plan to reform the program. A key element of this plan is to make Medicaid more effective and efficient by developing policies that will "maintain or even [improve] health outcomes while potentially saving money for both the states and the federal government" (6). One way to improve the health of Medicaid recipients and potentially reduce the rate of growth in Medicaid program expenditures is by covering PHS-recommended treatments, including individual and group telephone counseling and approved drugs (9,21-24).

### Strengths and limitations

The MEPS-NHIS national model that was used to calculate our state-level estimates is an improvement on a previous study that used data from the 1987 National Medical Expenditure Survey (NMES) to estimate smoking-attributable Medicaid expenditures (11). Results from the 1987 NMES are dated, and unlike NHIS, many of the key smoking variables that NMES used were imputed (25). Using recent data and actual, as opposed to imputed, smoking information in our calculations provides states with updated and accurate information that may better inform policy decisions. In addition, these differences may, in part, explain why the nationwide Medicaid smoking-attributable fraction of 11.0% is more conservative than the previous estimate of 14.5% generated for 1993 (11). Other changes that may account for the difference in our estimated smoking-attributable fraction include potential changes in the number of people treated for smoking-related illness from 1993 to 2002 and any change in treatment disposition from inpatient to outpatient care. Finally, our estimates differ from previous estimates, and probably understate Medicaid smoking-attributable expenditures, because they exclude expenditures for nursing home care, which are not available in the MEPS-NHIS model.

Despite these strengths, our study has several limitations. First, both the MEPS-NHIS and BRFSS are limited to noninstitutionalized populations, but we apply the resulting smoking-attributable fractions to expenditure estimates that include both institutionalized and noninstitutionalized populations. If these fractions are different for the institutionalized population, our expenditure estimates would be biased. Second, data limitations precluded us from quantifying smoking-attributable medical expenditures for smokers younger than 18 years and nonsmokers exposed to secondhand smoke. The effects of secondhand smoke on children's health are considerable (7). Secondhand smoke exposure can lead to acute lower respiratory infections, such as bronchitis and pneumonia in infants and young children, and can cause children who already have asthma to experience more frequent and severe attacks (26). Although health care expenditures attributable to secondhand smoke exposure may be high, quantifying these expenditures is difficult. As a consequence, our estimates understate smoking-attributable expenditures. Third, our analysis is limited to health care expenditures and therefore does not address other expenses (eg, disability, decreased productivity, absenteeism) that result from smoking (7). Finally, because our focus was not to test statistically whether smoking-attributable expenditures were larger in some states than others, we did not calculate standard errors at the state level.

### Conclusions

An estimated 443,000 Americans die prematurely each year as a result of smoking or exposure to secondhand smoke (27). Medicaid recipients are disproportionately affected by tobacco-related disease because their smoking prevalence is approximately 53% greater than that of the overall US adult population (10). In addition to the individual health toll, the disproportionately higher smoking prevalence among Medicaid recipients imposes substantial costs on society. We estimate that smoking accounts for approximately 11% of Medicaid program expenditures. To improve the health of Medicaid recipients and potentially reduce the growth rate of expenditures, Medicaid programs in all 50 states and the District of Columbia are encouraged to follow the 2000 PHS guidelines and cover all recommended tobacco-dependence treatments and approved medications (18). The cost-effectiveness of these programs, combined with the high cost of smoking, suggests that such coverage may provide cost savings to the financially strapped Medicaid programs.

## Figures and Tables

**Table 1:** Characteristics of Adult MEPS-NHIS (2001 and 2002) and BRFSS (1998-2000) Medicaid Recipients With Data on Smoking Status^a^

Characteristic	MEPS-NHIS		BRFSS	

	Nonsmokers (n = 768)	Ever Smokers (n = 820)	Nonsmokers (n = 7,701)	Ever Smokers (n = 8,500)
**Sex**				
Male	21	33	23	32
Female	79	67	77	68
**Race/ethnicity**				
White	32	60	32	58
Black	34	23	28	21
Hispanic	26	12	35	17
Asian	6	2	3	1
Other	1	3	1	3
**Mean age, y**	36	40	36	38
**Region of residence**				
Northeast	20	19	36	29
Midwest	21	24	11	18
South	35	38	28	28
West	24	18	25	25
**Weight category**				
Underweight	2	3	3	3
Normal	24	31	33	37
Overweight	36	31	29	30
Obese	36	34	30	26
Missing data	2	1	6	3
**Education**				
Less than high school graduate	35	34	33	38
High school graduate	56	58	61	58
College graduate	9	8	6	4
**Marital status**				
Married	34	24	37	32
Widowed	4	3	5	4
Divorced/separated	24	35	18	27
Single	39	38	40	37

Abbreviations: MEPS, Medical Expenditure Panel Survey; NHIS, National Health Interview Survey; BRFSS, Behavioral Risk Factor Surveillance System.

a All data are percentages, except age.

**Table 2:** Four-Part Model Regression of the Effect of Smoking on Annual Medical Expenditures

Variable	Correlation (Standard Error)			

	Probability of Positive Expenditures	Probability of Positive Inpatient Expenditures	Logged Expenditures for Users of Inpatient Services	Logged Expenditures for Nonusers of Inpatient Services
**Intercept**	4.19 (1.62)	−1.51 (1.21)	9.39 (0.80)	5.41 (0.70)
**Smoking status**				
Nonsmoker	Reference	Reference	Reference	Reference
Ever smoker	0.06 (0.24)	0.22 (0.14)	0.13 (0.11)	0.05 (0.12)
**Weight category**				
Underweight	0.06 (0.89)	0.35 (0.56)	0.64 (0.51)	0.45 (0.38)
Normal weight	Reference	Reference	Reference	Reference
Overweight	−0.08 (0.27)	−0.24 (0.27)	−0.16 (0.20)	−0.04 (0.16)
Obese	0.28 (0.26)	0.34 (0.26)	−0.02 (0.20)	0.21 (0.13)
Missing data	−0.88 (0.48)	−1.71 (0.72)	0.62 (0.22)	0.79 (0.34)
**Sex**				
Male	Reference	Reference	Reference	Reference
Female	0.81 (0.24)	−0.29 (0.24)	0.01 (0.16)	0.33 (0.18)
**Race/ethnicity**				
White	Reference	Reference	Reference	Reference
Black	−0.79 (0.30)	−0.34 (0.22)	−0.26 (0.16)	−0.57 (0.18)
Hispanic	−0.85 (0.28)	−0.08 (0.26)	−0.19 (0.13)	−0.55 (0.17)
Asian	−1.17 (0.54)	−0.72 (0.63)	−0.76 (0.35)	−0.85 (0.39)
Other	−0.96 (0.70)	−0.26 (0.59)	0.59 (0.36)	0.62 (0.30)
**Age**	−0.22 (0.10)	−0.04 (0.06)	−0.01 (0.04)	0.01 (0.04)
**Age squared**	0.00 (0.00)	0.00 (0.00)	−0.00 (0.00)	0.00 (0.00)
**Region of residence**				
Northeast	Reference	Reference	Reference	Reference
Midwest	−0.22 (0.40)	0.17 (0.28)	0.23 (0.17)	0.14 (0.25)
South	−0.33 (0.33)	0.37 (0.24)	0.10 (0.15)	0.19 (0.20)
West	0.12 (0.31)	−0.17 (0.28)	0.20 (0.20)	0.09 (0.21)
**Education**				
Less than high school diploma	Reference	Reference	Reference	Reference
High school diploma	0.37 (0.22)	0.18 (0.19)	−0.03 (0.12)	0.15 (0.12)
College	0.87 (0.65)	0.06 (0.31)	−0.21 (0.24)	0.03 (0.25)
**Marital status**				
Married	Reference	Reference	Reference	Reference
Widowed	0.44 (0.77)	0.28 (0.48)	0.24 (0.28)	0.71 (0.33)
Divorced/separated	1.30 (0.30)	−0.05 (0.21)	0.07 (0.16)	0.24 (0.13)
Single	0.35 (0.22)	−0.09 (0.21)	0.01 (0.14)	0.19 (0.14)
**Pregnancy**				
Not pregnant	Reference	Reference	Reference	Reference
Pregnant	3.67 (1.09)	3.77 (1.17)	−1.69 (0.59)	−0.64 (0.54)
***R* ^2^**	0.10	0.13	0.21	0.17

**Table 3:** Smoking Prevalence and Estimated Fraction and Total Annual Medicaid Expenditure Attributable to Smoking, by State

**State**	**Smoking Prevalence, %**	**SAF, %^a^**	**SAE, million, 2004 $**
Alabama	52	9	285
Alaska	68	15	67
Arizona	49	18	377
Arkansas	54	11	167
California	45	11	2,254
Colorado	61	17	338
Connecticut	49	7	249
Delaware	58	10	55
District of Columbia	51	11	95
Florida	46	11	951
Georgia	42	10	372
Hawaii	62	11	69
Idaho	62	14	97
Illinois	58	11	905
Indiana	68	15	521
Iowa	61	10	166
Kansas	54	12	171
Kentucky	65	12	390
Louisiana	43	12	364
Maine	63	14	190
Maryland	51	12	386
Massachusetts	53	11	696
Michigan	64	13	727
Minnesota	54	11	423
Mississippi	35	9	197
Missouri	66	14	514
Montana	70	15	70
Nebraska	64	15	167
Nevada	62	11	66
New Hampshire	80	15	103
New Jersey	36	6	309
New Mexico	50	12	159
New York	54	11	3,343
North Carolina	63	11	622
North Dakota	63	12	53
Ohio	65	13	1,171
Oklahoma	58	12	233
Oregon	67	15	290
Pennsylvania	70	11	849
Rhode Island	48	8	94
South Carolina	41	11	336
South Dakota	69	16	68
Tennessee	58	11	443
Texas	43	11	987
Utah	54	14	149
Vermont	67	15	74
Virginia	58	11	294
Washington	67	18	464
West Virginia	67	11	180
Wisconsin	63	13	440
Wyoming	62	16	40
US total	51	11	21,951

Abbreviations: SAF, smoking-attributable fraction; SAE, smoking-attributable expenditure.

a Estimates for states are based on Behavioral Risk Factor Surveillance System state-representative data and the Medical Expenditure Panel Survey and National Health Interview Survey (MEPS-NHIS) national model. The fraction for the United States as a whole is based solely on the MEPS-NHIS national model.
